# Redefining pandemic resilience: a roadmap for post-infectious syndrome preparedness and health system transformation

**DOI:** 10.3389/frhs.2026.1779647

**Published:** 2026-06-10

**Authors:** Bernhard Schieffer

**Affiliations:** Department of Cardiology, Angiology and Critical Care Medicine, University Hospital Marburg (UKGM), Marburg, Germany

**Keywords:** COVID - 19, digital health, healtch care system resilience, pandemic prepardness, postCOVID syndrome

## Abstract

Post-infectious syndromes like Long COVID and ME/CFS lead to disability and economic losses globally, especially in lower-income countries. These involve complex multisystem issues such as immune disturbances, inflammation, autonomic dysregulation, vascular problems, altered metabolism, and tissue damage. Re-infections increase the risk of disability and complications. Healthcare delays diagnosis and neglects long-term effects. We propose a three-part healthcare approach: primary care screening with digital tools, regional testing centers, and specialized Centers of Excellence for complex cases. An integrated infrastructure with registries, patient data, and wearables supports personalized care and surveillance. Policies should include disability benefits, rehab, infection control, and innovative funding. Healthcare must be accessible via mobile and community efforts, integrated into pandemic plans. The goal is to reduce morbidity and improve socioeconomic resilience.

## Introduction

### Burden and economic impact

Over the past century, major public health crises have repeatedly reshaped global health systems, highlighting the evolving relationship between human societies and infectious pathogens. The 1918 influenza pandemic, for example, demonstrated the catastrophic consequences of uncontrolled pathogen transmission and prompted the formation of innovative international epidemiological collaborations. These historical events underscore that resilience in public health depends not only on scientific progress but also on our collective capacity for humility, adaptability, and strategic foresight in confronting the inherent unpredictability of microbial threats (1). Subsequent milestones, including the post-World War II era and the HIV/AIDS epidemic in the late 1980s, further strengthened international collaboration, advanced chronic disease management and investment in translational research, and enabled countries such as the UK, Germany, and the US to establish modern public health systems (2, 3).

Recent outbreaks, including SARS-CoV-1, Middle East Respiratory Syndrome (MERS), and especially COVID-19, have revealed vulnerabilities in health systems. COVID-19 has highlighted the need for integrated surveillance, translational research, and adaptable health systems. Countries such as Germany, the UK, and the US lead in understanding these diseases (4). However, history shows a cycle of crisis-driven innovation followed by complacency, leading to preparedness gaps. To break this cycle, lessons must be institutionalized and global cooperation sustained, with an emphasis on international collaboration (1).

Post-infectious syndromes such as Long COVID and ME/CFS, which emerged after COVID-19, significantly contribute to the global disease burden and have complex medical and socioeconomic impacts. These conditions are underestimated worldwide, especially in low- and middle-income countries with limited healthcare (5–7). Despite advances in pandemic response, their chronic and disabling effects are underrecognized in clinical and policy frameworks (8). Evidence shows increased risks of neurodegenerative, autoimmune, and cardiovascular disorders after SARS-CoV-2, involving immune dysregulation, chronic inflammation, autonomic dysfunction, and tissue injury (5). Each reinfection may heighten the risk of long-term disability, with 5%–8% developing Long COVID. Ignoring post-viral effects could be costlier than immediate pandemic responses. Millions in Europe and North America face long-term work disability, worsening labor shortages, and increasing social costs. In Germany alone, hundreds of thousands are affected, causing billions in lost productivity. Addressing these sequelae is crucial for economic recovery, as failure to do so reduces workforce capacity and productivity (9–11).

### Areas for improvement in existing healthcare systems

Current policy and healthcare frameworks lag behind the growing complexity of post-infectious syndromes. In many countries, inconsistent recognition across insurance and disability systems creates unequal access to benefits and exacerbates social disparities. Policymakers should prioritize public health reforms to ensure equitable care. Despite global focus, national responses remain inadequate to address COVID-19's long-term health and economic effects (12, 13). Ongoing uncertainty complicates care delivery because of limited understanding, diverse patient symptoms, and a lack of validated biomarkers, which hinder diagnosis, treatment, and planning. While translational research and multi-omics studies offer hope, progress is slow (9).

Digital health technologies such as AI-based analytics and electronic health records can enhance biomarker discovery, real-time symptom tracking, and personalized care. Yet, fragmented use, low interoperability, and access issues weaken their impact, especially in resource-poor areas. Solving this requires ongoing interdisciplinary efforts that connect research, clinical care, social systems, and policy. Key priorities include cohort studies, expanding competence centers, monitoring chronic conditions, and prevention through improved indoor air quality, infection control, and workplace measures and accommodations (14–16). International collaboration through standardized data-sharing and preparedness frameworks remains essential for mitigating long-term health and socioeconomic impacts (4, 17).

## Discussion

### Reframing pandemic resilience

Redefining pandemic resilience involves shifting from short-term crisis response to long-term reduction of disease burden and support for socioeconomic stability. This requires better health infrastructure, coordinated care, and ongoing surveillance. Specialized care lessens healthcare strain and improves understanding of chronic inflammatory and neuroimmune disorders (18).

**Figure 1:**
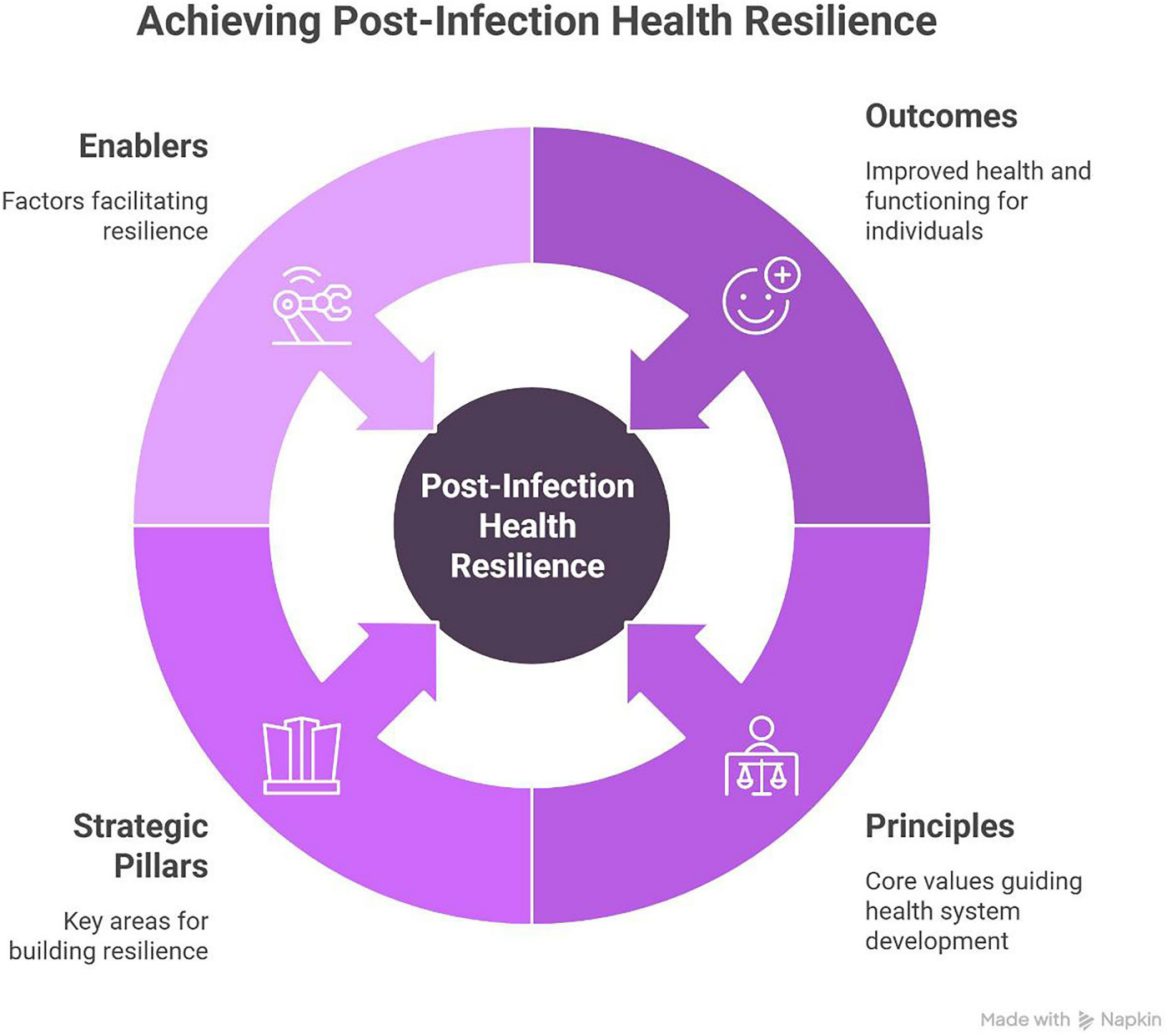
Summary of the core components contributing to post-infection health resilience.

**Figure 2:**
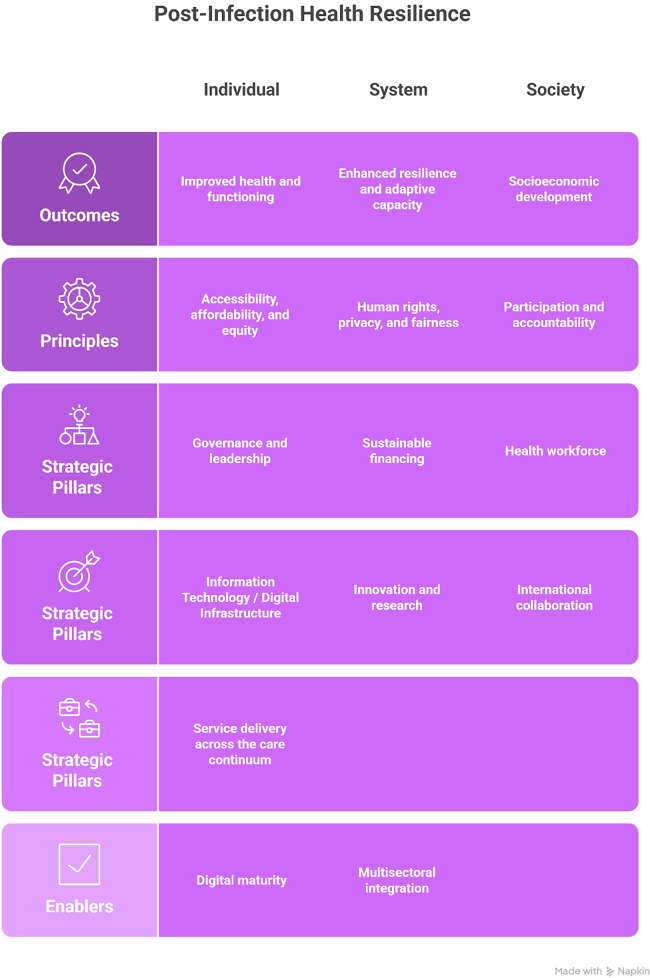
Strategic pillars for post-infection health resilience: this framework aims to improve pandemic preparedness and health system resilience. It includes seven pillars: governance and leadership, sustainable financing, health workforce, digital infrastructure, innovation and research, international collaboration, and service delivery. Each pillar covers key functions like diagnostic capacity, workforce training, resource distribution, biomarker research, data systems, antimicrobial stewardship, governance, and global coordination. These pillars help address the health, social, and economic impacts of post-infectious syndromes such as Long COVID and ME/CFS, underscoring the need for coordinated, multisectoral action.

To address how we might achieve post-pandemic health resilience, a strategic roadmap incorporating four core components: outcomes, principles, strategic pillars, and enabling conditions, as shown in Figure 1, should be adopted. Figure 2 visualizes the required measures on the individual, systemic, and societal level. At the individual level, the aim is to improve health status, functional capacity, and quality of life; at the health-system level, to build integrated care pathways and adaptive capacity; and at the societal level, to reduce disability, preserve workforce participation, and sustain economic stability. This transformation should be guided by equity, affordability, human rights, privacy, fairness, participation, and accountability. These principles must be embedded in legislation, financing, procurement, implementation, and evaluation to ensure that preparedness remains inclusive, rights-based, and responsive to patients and communities. Operationally, countries should align governance, sustainable financing, workforce development, digital infrastructure, research, and international collaboration to support care across the full continuum. Digital systems and multisectoral cooperation enable data integration, service coordination, and adaptability to evolving conditions. Implementation must be context-sensitive, negotiated nationally, and tailored to resources and needs (19, 20).

### Legal and ethical requirements

Building on guiding principles, this section establishes the legal, ethical, and governance foundations for their operation. Sustainable health policy must be grounded in a comprehensive legal and ethical framework that meets constitutional obligations and fosters patient and community participation. In modern health systems, responsibilities include digital health governance and data management. State obligations to protect safety, prevent discrimination, and promote welfare require clear authority structures, accountability, and legally embedded reforms (20). Pandemic preparedness should address long-term mortality and morbidity and ensure equitable access to diagnostics, treatment, rehabilitation, and social support. Ethical implementation depends on privacy-by-design, robust data security, equitable access policies, and research standards, including consent, risk assessments, and data management (4, 21). Transparent resource allocation that considers equity and social determinants of health is essential, as is alignment with international standards for multinational coordination (20). Governance and capacity are essential to effective implementation, including interoperable data sharing, standardized protocols, and investments in digital infrastructure. Capacity-building includes training policymakers on coordination and equity, clinicians on diagnostics and referrals, and the workforce on digital health literacy (21, 22). Community participation is crucial for accountability and inclusiveness, as highlighted in the guiding principles.

### Roadmap for strategic implementation

Despite extensive research efforts, biological complexity and diagnostic uncertainty in post-infectious syndromes hinder the development of therapies. A coordinated strategy is needed to implement the strategic pillars by translating them into priorities across governance, financing, workforce, digital infrastructure, research, collaboration, and integrated services (5, 7, 23). Table 1 provides an overview for the following strategic pillars.

(1) Governance and Leadership must ensure effective management characterized by accountability, equity, and adaptability. Key actions involve legally acknowledging post-infectious syndromes within public health and disability frameworks, ensuring equitable access to diagnostics and care, both legally and practically, and incorporating participatory governance that includes patient and community input. Additionally, governments should enhance their adaptive management skills through contingency planning and continuous policy review to address emerging health threats (19, 20).
(2) Sustainable Financing is essential for ensuring long-term system resilience and relies on a variety of scalable funding sources. Blended funding models that combine public funds, innovative financing techniques, and public–private partnerships can support infrastructure development, biomarker research, clinical trials, and the expansion of digital health. By pooling resources and focusing on outcome-oriented investment strategies, we can encourage equitable advancement while safeguarding financial stability and sustainability (15, 24).
(3) A well-trained and supported health workforce is essential for delivering quality and consistent care. Workforce strategies should prioritize training clinicians and policymakers in standardized diagnostics, interdisciplinary care pathways, digital skills, and cultural competence. Continuous professional development, enhanced by international knowledge exchange and clinical networks, can improve adaptability and ensure health systems remain responsive to evolving scientific evidence and patient needs (25–27).
(4) Digital infrastructure and information technology are essential for a strong health system, comprising secure and interoperable digital ecosystems. Important steps include employing AI-driven analytics, interoperable electronic health records, and remote monitoring devices to facilitate early detection and continuous care. In resource-limited settings, mHealth solutions and supervised task-shifting can expand access. Digital cognitive behavioral therapy (CBT) and telerehabilitation, when customized and carefully supervised, can help prevent symptom worsening (28, 29). Privacy-centric data sharing and federated analytics are vital for long-term monitoring and the development of learning health systems. Integrated digital platforms can accelerate diagnosis, customize treatment plans, and support real-time population health tracking (14, 25).
(5) Innovation and research should focus on discovering advanced biomarkers and developing therapies by leveraging multi-omics cohorts, systems biology, and integrating practical clinical trials into routine care. The use of sophisticated computational methods, including machine learning, mechanistic modeling, and digital algorithms, can help with disease classification, prediction of treatment outcomes, and the design of optimal rehabilitation strategies. Ensuring transparent modeling workflows, equitable data sharing, and standardized reporting is crucial for fairness, reproducibility, and relevance to effective policymaking (23, 25–27, 30).
(6) Global cooperation is essential to address the international burden of post-infectious syndromes globally. Countries should promote standardized data sharing, coordinated research, and cross-sector collaborations across clinical, public health, digital, and social systems (31, 32). Adjusting care models for low- and middle-income settings, including community-based care, supervised task-shifting, and mobile health solutions, can help promote equitable global healthcare implementation (7).
(7) Post-infection health resilience requires integrated services across prevention, detection, treatment, and rehabilitation. Prevention efforts include hygiene, vaccination support, and antimicrobial stewardship. Early detection depends on pathogen monitoring, diagnostics, and digital alerts for rapid responses. Clinical care should be multidisciplinary, patient-focused, with standardized diagnostics and referrals. Rehabilitation within primary and community care supports recovery, reduces disability, and maintains social and workforce participation trajectory (4).

**Table 1:** Summarizes proposed strategies across pillars like governance, financing, health workforce, technology, innovation, international collaboration, and service delivery. These aim to build resilient, equitable health systems to address long-term effects of post-infectious syndromes such as Long COVID and ME/CFS. Each action includes its expected impact to aid policy planning, implementation, and evaluation.

Strategic pillars	Key Goals	Options for Action
Governance and Leadership	Guarantee ongoing support and accountability.	Legally recognize post-infectious syndromes as disabilities and embed resilience in public health law.
	Enhance adaptive management capacity.	Ensure fair and equal access (de jure and de facto)
		Adapt to emerging threats, develop contingency plans.
Sustainable Financing	Ensure long-term viability and scalability.	Use blended public investment, innovative financing, and public-private partnerships.
Health Workforce	Improve the quality and consistency of care through a properly trained health workforce.	Train clinicians and policymakers in standard diagnostics, digital skills, and cultural competence
Information Technology/Digital Infrastructure	Developing interoperable digital infrastructure	Implement AI-enabled analytics, interoperable EHRs, and remote monitoring for early detection and care.
		Utilizing long-term surveillance and data sharing
		Accelerate diagnosis, personalize care, and enable real-time surveillance.
Innovation and Research	Advance biomarker discovery and therapy development	Embed multi-omics research and pragmatic trials into routine care and policy
		Using digitalized mathematical algorithms and technologies
		Fractal/multifractal approaches
International Collaboration	Facilitate rapid knowledge transfer and equitable preparedness	Standardize data-sharing and preparedness protocols; invest in global capacity
		Adapting models for low- and middle-income countries (LMICs)
Service delivery across the care continuum	Infection control and prevention	-Implement hygiene protocols
		-Support vaccination
		-Improve antimicrobial stewardship
	Early detection and surveillance	-Improve pathogen monitoring
		-Enhance diagnostic capabilities
		-Create alert systems
	Clinical care	-Establish standardized diagnostic and referral pathways
		-Develop multidisciplinary care models integrating primary, specialist, and mental health services
		-Implement evidence-based clinical guidelines and decision-support tools
	Rehabilitation	-Develop individualized, multidisciplinary rehabilitation programs tailored to symptom variability.
		-Expand access to community-based and home-based rehabilitation models
		-Implement digital rehabilitation and remote monitoring where appropriate
		-Support return-to-work programs and social reintegration strategies

### Implications for policy and practice

The COVID-19 pandemic revealed weaknesses in global health systems, especially their inability to handle long-term effects of post-infectious syndromes like cognitive impairments, autonomic dysfunction, and chronic fatigue, which cause economic and social issues such as workforce shortages and health disparities (18). While response speed has improved, many systems remain reactive, leaving millions without adequate care, recognition, or support. To build resilience, health systems must adopt proactive, integrated approaches that integrate surveillance, digital health, and research into policies and clinical practice. This includes scalable surveillance, biomarkers, multidisciplinary care, and accessible rehabilitation to support recovery and maintain economic and social stability (14).

These efforts require international cooperation, standardized data collection, and ethical implementation frameworks to institutionalize lessons learned and prioritize vulnerable groups. Resilient health systems should not only prepare for future crises but also predict, mitigate, and adapt to ongoing impacts, embracing innovation, equity, and foresight to protect health, sustain economies, and serve society now and in the future (4). Therefore, advancing post-pandemic care depends on integrating digital technology, multi-omics data, and modeling approaches. Digital twins, or virtual patient models, combined with genomic, physiological, environmental, and lifestyle data, enable clinicians to simulate disease and forecast treatments in real time. AI-based models enable risk assessment, phenotype categorization, and the development of adaptable therapies by analyzing health data. These tools accelerate biomarker discovery, support symptom tracking, and improve clinical workflows, aiding healthcare and policy. Embedding clinical trials within learning health systems can accelerate biomarker research and the adoption of digital therapeutics. Advances require strong ethical, legal, and governance frameworks to protect privacy, ensure transparency, and promote equity, especially in resource-limited settings. Sustainable funding and international cooperation are vital for global impact. These trends shift care from reactive to proactive, predictive, and participatory, learning and adapting to reduce long-term illnesses and strengthen resilience. This review is intended as a conceptual, policy-oriented synthesis, not a systematic comparative analysis. While the proposed roadmap builds on evolving evidence about post-infectious syndromes and health system resilience, many components lack validation across diverse contexts. Variations in healthcare infrastructure, workforce capacity, digital maturity, governance, and fiscal space affect transferability. Resource-intensive elements such as diagnostics, data systems, multidisciplinary centers, and advanced tools depend on countries' financing, reimbursement, and budgets. The framework should be adaptable, calibrated to local epidemiology, capacities, and financial resources. For low- and middle-income countries, prioritized, phased, and community-based approaches, along with task-sharing strategies and mobile health, may be more feasible. Future research should analyze implementation, feasibility, and cost-effectiveness to identify the most beneficial elements across different health system settings.

This review summarizes a strategic framework that links early recognition, multidisciplinary care, rehabilitation, surveillance, digital infrastructure, and research to enable a coordinated response. Its aim is to enable earlier diagnosis, equitable care, better outcomes, and socioeconomic resilience. Implementation should be phased and context-sensitive, depending on each country's capacities. Future research will refine its applicability across diverse health systems. Key factors include immune, autonomic, endothelial, and metabolic dysfunctions. Transitioning to a *Three-Tiered Care Pathway* from primary screening to Centers of Excellence with integrated diagnostics and multidisciplinary management is recommended. Developing a Learning-Health Infrastructure with data-driven, privacy-focused, personalized care and supporting policy and funding through coordinated benefits, scalable rehab, and sustainable resources is crucial. Emphasizing equity and flexibility will ensure global applicability, including resource-limited settings.

## Data Availability

The original contributions presented in the study are included in the article/Supplementary Material, further inquiries can be directed to the corresponding author.
